# Organisation, influence, and impact of patient advisory boards in rehabilitation institutions—an explorative cross-sectional study

**DOI:** 10.1186/s12891-022-05678-y

**Published:** 2022-08-02

**Authors:** Joachim Sagen, Elin Børøsund, Ann Elisabeth Simonsen, Andreas Habberstad, Ingvild Kjeken, Hanne Dagfinrud, Rikke Helene Moe

**Affiliations:** 1 Oslo Metropolitan University, St. Olavs plass, P.O. Box 4, NO-0130 Oslo, Norway; 2 Department of Digital Health Research, Division of Medicine, Oslo University Hospital, Oslo, Norway; 3 Røysumtunet Rehabilitation Center, Jaren, Norway; 4 The Norwegian Federation of Organisations of Disabled People, Oslo, Norway; 5 Norwegian National Advisory Unit On Rehabilitation in Rheumatology, Oslo, Norway

**Keywords:** Patient engagement, Patient participation, Health care services, Rehabilitation, Organisational, Institutional, Meso-level, System-level

## Abstract

**Background:**

Patient participation is highlighted as an important facilitator for patient-centered care. Patient participation organised as patient advisory boards (PABs) is an integral part of health care institutions in Norway. More knowledge is needed on how PAB representatives experience patient engagement (PE) with regard to organisation, influence, and impact. The objective was to describe how PAB representatives experience their tasks, roles, and impact on decision-making processes and service delivery in the setting of rehabilitation institutions.

**Methods:**

PAB representatives recruited from rehabilitation institutions completed the Norwegian version of the generic Public and Patient Engagement evaluation tool (Norwegian abbreviation EBNOR). EBNOR is tested for reliability and validity with good results and comprises 35 items within four main domains, policies and practices, participatory culture, collaboration, and influence and impact that provide responses about PE-levels. The domain items are scored from “strongly disagree” to “strongly agree” on a five-point scale, in addition to a don’t know category. Items in the domain “influence and impact” are scored from “never” to “all of the time” on a four-point scale. Categorical data were summarized using frequencies and percentages, and response categories were collapsed into three PE-levels: barrier, intermediate, and facilitating level. Free-text responses were analysed according to principles of manifest content analysis, summed up, and used to elaborate the results of the scores.

**Results:**

Of the 150 contacted PAB representatives, 47 (32%) consented to participate. The results showed that approximately 75% agreed that the organisation as a whole was strengthened as a result of patient participation. Four out of five domains were scored indicating a facilitating level; policies and practices (53%), participatory culture (53%), collaboration and common purpose (37%), and final thoughts (63%). The modal score in the domain influence and impact was in the intermediate PE-level (44%). Of a total of 34 codes from free text analyses, barriers to PE were coded 26 times, and PE facilitators were coded 8 times.

**Conclusions:**

The findings indicate that most PAB representatives are satisfied with how rehabilitation institutions organise their PAB, but they still experience their impact as limited.

**Supplementary Information:**

The online version contains supplementary material available at 10.1186/s12891-022-05678-y.

## Background

Rheumatic and Musculoskeletal Disease (RMD) is the most common cause of disability in Europe and is described as one of the major challenges to human health [1]. About 75% of years lived with disability are due to conditions for which rehabilitation may be beneficial [2], and WHO calls for action to address these health needs [3]. In Norway, RMDs are the single largest diagnostic group in need of rehabilitation, about 30% of patients who receives in- or outpatient services in private rehabilitation institutions are due to RMDs [4]. Engagement of and participation by patients may serve as an important key to meeting the current and future health care needs of people with RMDs [1].

Over the last decades, patient-centered care has become increasingly prioritized at all levels of health care services [5]. A patient-centered health care service advocates patients’ needs and values, both in the health care setting and in everyday life, considering each person’s health-related knowledge [6, 7]. It is argued that patient-centered care has the potential to improve self-dignity, quality of life, well-being, and relevance of care, as well as to reduce costs [8, 9].

The shift towards a more patient-centered health care service has increased the focus on individual rights driven by engaged patient organisations and political incentives, resulting in a shift from institutionalization and paternalism to democratization and individualization [10]. Patient-centered care can be facilitated by active participation from patients and patient representatives, defined as patient engagement (PE) [11, 12]. Even if PE can facilitate patient-centered care, there is still little data supporting that PE has an impact on health care delivery [13].

Patient participation can take place at micro, meso, or macro-levels [14–16]. At the macro-level, patient representatives participate in shaping national health care policy. The micro-level refers to patients participating in shared decision-making regarding their own treatment. In recent years, patient participation in the development and delivery of health care services at the meso-level has been highlighted as important [17]. At this level, patient representatives use their unique expertise to influence how planned actions are designed, organized, and/or delivered to meet the needs of larger groups of patients [8, 18].

Today, national policies around the world call attention to patient-centered health care services [19]. However, there is limited agreement on how to engage patient representatives in a meaningful manner [20]. Various stakeholders display dissimilar views on what patient participation should entail according to values, roles, and organisation [21]. In some cases, there are indications that formal meso-level patient participation is performed as tokenism, and is thus not founded on mutual respect, or rooted in organisational culture [21, 22].

In Norway, political strategies state that patient participation constitutes one of six dimensions that define the quality of the health care service [23]. The National Health Plan 2020–2023 stipulates that patients should be involved in shaping all parts of their health care service [24]. Although patient participation is a contributor to quality, it is an area with great potential for improvement [25]. There are major differences in how patient participation in health care services at various levels of care is organised and described related to patient representative’s role and influence [26].

To meet political demands and formally address patient-centered care, patient advisory boards (PABs) are included as a statutory part of rehabilitation institutions in Norway. Even so, there are no general rules or procedures regarding how PABs should operate, including how PAB representatives are recruited. Therefore, there is a need to explore how PAB representatives experience their organisation, influence and impact. As rehabilitation institutions implement PABs, rigorous evaluation of PAB participation is needed to ensure future quality in meso-level PE.

This study aims to explore the impact of PE and patient participation in rehabilitation institutions. More specifically, the objective was to describe how PAB representatives experience their tasks, roles and impact on decision-making processes and service delivery in the setting of rehabilitation institutions.

## Method

### Design

The study was performed using an exploratory, cross-sectional design.

### Data collection

A web-based survey was distributed by e-mail, provided by Quest Back. Recruitment and data collection took place between February and May 2021.

### Setting

The study was performed during a period when COVID-19 affected health care and rehabilitation. Meso-level patient participation was addressed by exploring a specific group of individuals working together in PABs. Participants in this study share a common interest in the organisation, development, and delivery of rehabilitation, as part of health care. Study participants were recruited from PABs from 37 private rehabilitation institutions representing all four health regions in Norway. These institutions offer inpatient and outpatient rehabilitation to a variety of diagnostic groups, with people with rheumatic and musculoskeletal diseases being the largest group. They are committed by Norwegian health authorities to engage patients in service development and delivery.

### Survey instrument

The evaluation of PE is limited by a lack of reliable and valid outcome measures [27]. Therefore, the generic Public and Patient Engagement Evaluation Tool (PPEET) was applied to gain more knowledge about PE and participation at the meso-level [28]. PPEET was designed to compare PE across health care organisations and assess how PE is performed and experienced. The Norwegian version of PPEET, named *Evalueringsverktøy for Brukermedvirkning* (EBNOR) [29], was applied. EBNOR is a translated, back-translated, and culturally adapted patient-reported instrument. The original as well as the translated version have been tested for content validity and reliability with good results [28, 29].

### Measures

EBNOR comprises 35 items representing four core domains relating to different domains of PE-activity: “Policies and practices” (six items), “participatory culture” (ten items), “influence and impact” (seven items), and “collaboration and common purposes” (three items). There are also sections with “background questions” (five items) and “final thoughts” (four items). The respondents are asked to take a position on various statements in each domain. Response categories are measured on a scale with three to five response options, indicating the level of PE, and a “don’t know” response category. The items in four domains are scored from “strongly disagree” to “strongly agree” on a five-point scale. Items in the domain “influence and impact” are scored from “never” to “all of the time” on a four-point scale. One item in the domain “participatory culture” scores “yes”, “no” or “don’t know”, and “final thoughts” items do not have a “don’t know” category. At the end of each domain, there is an open space that offers the possibility for free text additions or comments. All participants were informed that none of the items in the survey were mandatory, and it was possible to return to previous items on earlier pages if needed.

### Participants and recruitment

The inclusion criteria were current PAB representatives aged 18 or older affiliated with rehabilitation institutions. Invitations to take part in this study were sent to the email addresses of 150 PAB representatives registered by the rehabilitation umbrella organizations VIRKE and UNICARE. The electronic survey was thus administrated to 150 email addresses using convenience sampling.

### Ethics

All participants signed an informed consent before anonymously completing the survey. Quest Back enabled two automatic reminders to those who did not respond after the first four weeks. The survey closed on May 18, 2021. Given that no health-related data were collected or generated by the survey tool, the study did not require formal ethical approval, but the survey was approved by the data protection officer at Diakonhjemmet Hospital (DS-00040). The study was conducted in line with the Helsinki Declaration and is part of the RehabNytte study NCT03764982.

### Analysis

Items with categorical responses were summarized separately for each domain using frequencies and percentages. Distributional plots were inspected. Response options were collapsed from the original three to five categories into three categories, plus the “don’t know” category as follows: strongly disagree and disagree, neither agree or disagree, and agree and strongly agree. The response categories “rarely” and “some of the time” were also collapsed. Item 14 had response options yes, no and don’t know, and was not collapsed. The collapsed categories were divided into PE-levels, presented as a barrier, intermediate, or facilitating level. Data were systematized using IBM SPSS version 27.

Free-text responses were analysed abductively and descriptively according to principles of manifest content analysis by two researchers (JS and EB) independently [30, 31]. All free-text responses were first read as a whole and then analysed answer by answer. Words or sentences were categorised into meaning units and labelled with codes using an agreed coding scheme. Themes and meaning units were reviewed by each researcher separately and then discussed by the research team until a consensus was reached. The codes were thereafter compared and sorted into one of two themes reflecting facilitators or barriers to PE related to the domains. Free text data were analysed using Quirkos version 2.4.1. The free-text responses were used to elaborate the results of the scores.

### Patient participation statement

In this study, patient representatives were actively involved as advisors and research partners from the planning phase. Both the Norwegian Federation of Organisations of Disabled People (FFO), a patient umbrella organisation, and a representative for PABs in rehabilitation, was engaged at an early stage and helped develop the main research priorities and interests. After the research topic was established and funding ensured, the two research partners participated in the discussion, interpretation, presentation and publication of the results. These two patient research partners contributed to the use of inclusive language in the final manuscript and to the plain language summaries in Norwegian (Additional file 1) and in English (Additional file 2), they were engaged in all stages of the research project. For a detailed description of how PPI were incorporated into the study design, please see Additional file 3 (GRIPP-2 checklist). All relevant patient research partners were included as authors in accordance with the Vancouver declaration.

## Results

All of the contacted institutions, except one, had an active operating PAB. A total of 150 eligible PAB representatives were contacted via email, 63 agreed to participate, and 47 (32%) of these responded to the survey (Table 1). Based on the age categories the PAB representatives’ mean age was approximately 60.5 (30 to 81 yrs.), and a majority (81%) had above two years’ experience with patient participation. Some of the PAB representatives (*n* = 9) reported to have an additional specific organisational role, for instance representing a patient organisation.

**Table 1:** Demographic characteristics of the study sample from the electronic survey (*N* = 47)

Respondents		
Demographics^a^	n	%
Age		
30–39	1	2
40–49	10	22
50–59	11	23
60–69	13	28
70–79	11	23
≥ 80	1	2
Gender		
Female	29	62
Highest completed education		
Basic (≤ 10 yrs.)	5	11
Secondary (11–13 yrs.)	10	21
College or university	32	68
User experience		
≤ 2 years	9	19
> 2 years	38	81
Health region		
North	4	9
West	2	4
Central region	10	21
South East	31	66
Organisational role		
Board member	3	7
Member of PAB	36	76
Employee	1	2
Senior manager	2	4
Patient organisation	3	7
Missing	2	4

^a^The percentages are rounded up or down to meet a total of 100

In general, when inspecting the plots, data were skewed towards a facilitating PE level, except for the domain *influence and impact*, in which the modal value (most frequent value) was at the intermediate PE-level (Table 2).

**Table 2:** Modal values of collapsed categories, divided into patient engagement levels [7]

		Barrier PE-level	Intermediate PE-level	Facilitating PE-level	
Domain/ Item^a,b^		Strongly disagree/disagree n (%)	Neither agree nor disagree n (%)	Agree/strongly agree n (%)	Don’t know n (%)
*Policies and practices *^c^					
6	Explicit strategy for PE^d^	4 (8)	5 (11)	**37 (79)**	1 (2)
7	Explicit strategies for recruiting participants	5 (11)	13 (28)	**28 (59)**	1 (2)
8	Identified resources for PE	5 (11)	9 (19)	**20 (42)**	13 (28)
9	Adequate PE resources	10 (21)	9 (19)	**18 (39)**	10 (21)
10	Prepares reports of PE	9 (19)	12 (26)	**22 (46)**	4 (9)
	**Total n (%)**	33 (14)	48 (20)	**125 (53)**	29 (13)
*Participatory culture *^e, f^					
12	Commitment to PE in key organisational documents	2 (4)	6 (13)	**35 (74)**	4 (9)
13	Commitment to PE through structure	8 (17)	11 (23)	**25 (53)**	3 (7)
15	Clear responsibilities for PAB^g^	6 (13)	8 (17)	**31 (66)**	2 (4)
16	Responsibilities in job descriptions of relevant staff	4 (9)	9 (19)	16 (34)	**18 (38)**
17	Comprehensive PE training/materials to support staff	11 (23)	10 (21)	**14 (30)**	12 (26)
18	Adequate PE training	7 (15)	13 (28)	**27 (57)**	0 (0)
19	Leaders show commitment to using PE input	3 (7)	10 (21)	**33 (70)**	1 (2)
20	Reports of contribution from PE shared with participants	5 (11)	17 (36)	**18 (38)**	7 (15)
	**Total n (%)**	46 (12)	84 (22)	**199 (53)**	47 (13)
*Influence and impact*^h^		Never	Rarely/some of the time	All of the time	Don’t know
22	PE contributions are identifiable	0 (0)	**28 (60)**	11 (23)	8 (17)
23	Leaders use input from PAB	0 (0)	**25 (53)**	13 (28)	9 (19)
24	Patient representatives are equal to employees in meetings	4 (9)	11 (23)	**29 (62)**	3 (6)
25	PAB representatives have voting rights in meetings with employees	7 (15)	10 (21)	13 (28)	**17 (36)**
26	Instances where PAB input had an influence	1 (2)	**30 (64)**	4 (9)	12 (25)
27	Instances where PAB input influenced management decisions	1 (2)	21 (45)	2 (4)	**23 (49)**
	**Total n (%)**	13 (5)	**125 (45)**	72 (25)	72 (25)
*Collaboration and common purpose*		Strongly disagree/disagree	Neither agree nor disagree	Agree/strongly agree	Don’t know
29	PE led to collaboration with other groups	5 (11)	10 (21)	**19 (40)**	13 (28)
30	PE led to identifying shared goals with other organisations	1 (2)	**16 (34)**	14 (30)	**16 (34)**
	**Total n (%)**	6 (6)	26 (28)	**33 (35)**	29 (31)
*Final thoughts*		Strongly disagree/disagree	Neither agree nor disagree	Agree/ strongly agree	
32	Appropriate level of engagement activity	9 (19)	12 (26)	**26 (55)**	
33	Appropriate level of resources to PE activity	14 (30)	10 (21)	**23 (49)**	
34	The organisation is strengthened as a result of PE	1 (2)	6 (13)	**40 (85)**	
	**Total n (%)**	24 (17)	28 (20)	**89 (63)**	

^a^The percentages are rounded up or down to meet a total of 100

^b^Modal values are presented in bold

^c^Question numbers 11, 21, 28, 31, and 35 are free text fields

^d^*PE* Patient engagement

^e^Items 6–10, 12–13, 15- 20, 29–30 and 32–34 valued 1–5: strongly disagree, disagree, neither agree nor disagree, agree, strongly agree, Items 32–34 did not have a don’t know category

^f^Item 14: valued 1–3: yes, no, don’t know, and was not collapsed and not presented in the table

^g^*PAB* Patient advisory board

^h^Items 22–27 valued 1–4: never, rarely, some of the time, all of the time and don’t know

The modal value regarding background items combined (items 1–5) was at the intermediate PE level, with approximately half of the responses. According to almost three quarters (74%) of the respondents, the institutions had established or were in progress of routinely engaging patient representatives in their activities. Of the respondents, 57% reported that they had some level of awareness of the institution overall PE approach, 28% had high level of awareness, and 15% were neither aware or unaware. Over 70% answered that they sometimes or fairly frequently interacted with professionals in charge of patient participation, and 2% interacted very frequently. Approximately 60% of free-text answers were coded as barriers (*n* = 26) or facilitators (*n* = 8) for PE. The number of codes with examples of condensed meaning units is presented in Table 3. The most frequent barrier was the direct or indirect *exclusion of PAB in institutional activities* (13 codes), and the most coded facilitator was the *inclusion of PAB* (4 codes). Regarding PAB exclusion, one participant stated: “One problem to genuine patient participation is use of hard-to-understand language in meetings, and too collegial attitude among health care professionals. I wonder if medical education is necessary to influence PAB processes”.

**Table 3:** Codes and themes of free-text responses^a^ with condensed example statements

Code	Frequency of codes	Theme	Examples using condensed meaning units
		Barriers to PE^b^	
Screening of information	2		“It appears that some information is adapted before made available to everyone.”
Unclear PAB tasks	1		“Unclear PAB mandate and role.”
Inaccessible information	3		“Reports from PAB should be open to all PAB representatives.”
Exclusion of PAB^c^ in institutional activities	13		“Pronounced use of hard-to-understand language.” “Leader of PAB does not have voting rights.” “Documents only available to employees.”
Resources dedicated to PE	2		“No resources are set aside for PAB education.”
Covid 19	5		“Not allowed to physically access the institution.”
**Total codes**^d^	**26**		
		Facilitating PE	
Inclusion of PAB in institutional activities	4		“Mostly quite open and clear dissemination.” “In some cases, it has been useful when employees have initiated PAB inclusion.”
Available information	2		“Reports are made available from PAB meetings.”
Covid 19	1		“Everything worked well and digital meetings have been arranged.”
Independence of PAB	1		“PAB has developed its guidelines and helped draw up ethical guidelines.”
**Total Codes**	**8**		

^a^Free-text answers (*N* = 30): Q11: practices and policies (*n* = 5), Q21 participatory culture (*n* = 7), Q28 Influence and impact (*n* = 8), Q 31 collaboration and common purpose (*n* = 3), Q 35 Final thoughts (*n* = 7)

^b^*PE* patient engagement

^c^*PAB* Patient advisory board

^d^Total codes related to a theme are presented in bold

For the domain *policies and practices* that includes items about PE strategies and resources, the modal value was at the facilitating PE level. Approximately 80% agreed or strongly agreed with the item *Do the organisation have an explicit PE strategy?* The free text item *resources dedicated to PE* contained answers that were coded two times as a PE barrier. One participant stated the following: “I am fairly new as a patient representative and have not seen an overview of financial recourses dedicated to patient participation”.

The domain *participatory culture* comprises items about PE commitment, responsibilities and stakeholder training. The modal value fell within the facilitating PE level for the domain. Over two-thirds of the participants reported that the institutions have available PE guidelines, such as values, principles, and responsibilities found in key organisational documents. Free-text answers indicated that the prioritization and organisation of patient participation changed as a result of the pandemic. One PAB representative stated: “I have not been allowed to access the institution since February 20, due to infection control”.

The domain *influence and impact* include items about identifiable PE contributions and equality among stakeholders. The modal value was placed at the intermediate PE level for the overall domain. For the item *equality in meetings*, the most frequent response category was found at the facilitating PE level, and for the item *voting rights* in the “don’t know category”. In relation to the item *voting rights*, one PAB representative stated: “I have the right to express my opinion, but not the right to vote”. Another stated: “I’m not sure if PAB representatives have the right to vote”. Regarding the item *PE input influenced management decisions*, the most frequent value was found for the “don’t know” response category. One PAB representative commented: “Sometimes patient participation is experienced more like information transfer than as mutual communication”.

For the domain *collaboration and common purpose*, which consists of two items about PE partnership with other organisations and shared goals, the modal value showed a facilitating PE level. The “don’t know” response category had the second-most frequent value.

Regarding the three final items about the level of PE activity, resources and perceived benefits from PE, the modal value indicated a facilitating PE level. One PAB representative specified: “Organisations with a focus on patient participation show that they do their best for the patients/…/the organisation is strengthened”.

## Discussion

This is one of very few studies to explore how PAB representatives experience their engagement in PABs and impact on decision-making processes and service delivery.

The results from this study mainly indicate a facilitating PE level. However, an important finding is that even if almost three-quarters of the participants reported that their institution had established or were in progress of routinely engaging patient representatives in their activities, a majority still experienced their impact as limited, seldom affecting institutional decisions. Thus, PE may not yet be an integral part of the culture of rehabilitation institutions. Findings by others may shed light on these contradictory results when describing lack of meaningful relations based on trust and respect as a PE barrier [32]. One reason may be that integrating PE as part of an institutions culture and structures may take time, and as time goes by, this will evolve as a natural part of service development. PE progress towards meso-level PAB impact may also be hindered by unwritten cultural values, experienced through structural assumptions and human interactions, referred to as “the glass ceiling” [33].

Even if meso-level patient participation within the field of rehabilitation is a relatively new focus area in Norway, this study indicates that a large majority of the respondents are aware of and included in the overall institutional PE approach, and have regular interactions with professionals in charge of patient participation. As suggested by others, this indicates a process in the direction of more participatory acceptance and shows institutional willingness to patient-initiated, meso-level impact [34]. Principles for carrying out high quality PE activities may progress towards procedural influence and meso-level impact by increasing the frequency of interaction between representatives and professionals. Recent findings suggest that the co-creation process itself may improve relations between PE stakeholders [35, 36].

A majority of respondents reported that a commitment to PE values and principles was to be found in key documents (74%) and stated in the organisational structure (53%). However, fully integrating PABs in institutional decisions by overcoming unwritten PE barriers is found to be an extensive process dependent on long-lasting commitment [32, 37]. Implementing agreement among PAB representatives and health care professionals in the setting of priorities can be seen in the context of allocated resources to facilitate PE. Involving patients and professionals in setting priorities has been found to require approximately 10 percent more time and 17 percent more financial resources than engaging solely health care professionals [27]. The results from our study may inform this finding, where the majority of participants did not agree or did not know if the organisation had enough resources for PE.

A majority (66%) reported that responsibilities related to PAB were clearly stated, and (62%) of the respondents answered that they felt equal to employees in meetings. Simultaneously, 32% did not have, or did not know if they had voting rights in these meetings, and only 4% could think of instances where PE had contributed to organisational change. The low proportion that regularly experienced meso-level impact can be understood by the presence of more or less invisible barriers such as to use of difficult language and withholding important information. Procedural experiences from PAB representatives that may contribute to the “The glass ceiling” are enlightened through the free-text responses. The lack of influence from PABs is exemplified by institutional changes due to the pandemic (Table 3). In contrast, free-text responses about the pandemic indicate that rehabilitation institutions have strengthened accessibility to attend meetings by arranging them digitally. Increased use of digital meetings may facilitate interaction among PE stakeholders. This may point to the positive potential of remote participation, with accelerated training and experience with digital tools, and with the potential of becoming regular PAB practice for rehabilitation institutions after the pandemic. Since the consequences of RMDs may include chronic pain, disability, social exclusion, and reduced productivity [1], the possibility of digital access to PABs may have the potential to strengthen the representation of this group of patients. In concordance with recent findings by others [38, 39], less time consuming and more accessible patient participation could ease the participation of PAB representatives regardless of the degree of functioning, disability or handicap, and thus strengthen representativeness.

Approximately half of the respondents could not think of events where input from PAB had consequences for management decisions. Regardless of a lack of procedural influence and structural impact identifiable for PAB representatives, roughly 75% agreed that the organisation as a whole was strengthened as a result of patient participation. Supported by findings from others [36, 40], this may imply that respondents see an intrinsic democratic value from being a part of the participatory process. Simultaneously, PABs may have a function as an administrative organisation for health care managers, who can register and show patient participation to control bodies, even if PAB contributions do not necessarily lead to meso-level impact.

The results of this survey indicate that PE may improve patient-centered rehabilitation and has the potential to benefit a large group of patients with RMDs. Active patient participation may optimize the organization of future rehabilitation, the collaboration between different stakeholders, and the management of RMDs. We identified better inclusion of PABs, and clearer PAB tasks as possible improvement areas to increase PAB impact and influence. To facilitate equal opportunities to influence development and delivery of rehabilitation services, institutions and institutional leaders should consider the importance of all relevant information to be understandable and accessible for PAB representatives. Rehabilitation institutions should also aim to clarify PAB tasks and responsibilities in agreement with PAB representatives before prioritizing further PE activities. Findings of this study imply that stakeholder adherence to PE values and commitments may facilitate active patient participation and should be prioritized. Active participation from professionals and PAB representatives may progress towards a patient-centered structure, facilitating equal opportunities to influence service delivery. A patient-centered structure could involve a commonly rooted reprioritization of resources, practices, and work tasks that are fully supported by all stakeholders in the PE process. However, there is a need to further explore how local and general political guidelines work as facilitators or barriers for PE and identifiable PAB contributions. Additional research is necessary to help explore factors that may increase PAB inclusion and for investigating how these factors may facilitate equal opportunities for influence and impact through active patient participation, and hinder tokenism.

### Limitations

This study has some limitations. First, due to the research aim, all of the participants were recruited from rehabilitation institutions in Norway. This may limit the generalization of results to other settings, health care systems and countries. Second, prolonged periods of unfamiliar operations within the rehabilitation institutions due to the pandemic may have led to challenges in generalizing the results to normal operations and circumstances. Third, there was a relatively small population of 150 PAB representatives, and the response rate was just above 32%, leaving the study prone to nonresponse bias. There is a possibility that participants who responded and commented were systematically more critical towards the health care services and/or more engaged than those who did not respond. The low response rate may have been amplified due to the pandemic, which in turn may have led to a reduced priority for patient representative-related tasks, such as responding to emails or questionnaires. Even so, most participants reported analogous representative PAB experiences.

## Conclusion

The findings of this study indicate that PAB representatives are generally satisfied with how rehabilitation institutions organise PABs, suggesting that most preconditions for meaningful co-creation are present. On the other hand, unwritten social PE barriers may affect PAB possibilities to influence meso-level decisions. The findings indicate that PAB representatives still experience their impact as limited. These results may contribute to future PAB participation with impact on patient-centered care.

## Supplementary Information

**Additional file 1.** Plain language summary in Norwegian.

**Additional file 2.** Plain language summary in English.

**Additional file 3.** PPI using GRIPP2 – SF.

## Data Availability

The data that support the findings of this study are available from the authors upon reasonable request and with permission of the research board.

## Abbreviations

PE: Patient Engagement
PAB: Patient advisory board
PPEET: Public and Patient Engagement Evaluation Tool
EBNOR: Evalueringsverktøy for Brukermedvirkning
