# Supplementary material for: Organisation, influence, and impact of patient advisory boards in rehabilitation institutions—an explorative cross-sectional study
Source: BMC Musculoskelet Disord. 2022 Aug 2;23:738. doi: 10.1186/s12891-022-05678-y (PMC9343240; doi:10.1186/s12891-022-05678-y)
Supplement: Supplementary file 1 — Additional file 1. Plain language summary in Norwegian. [file 12891_2022_5678_MOESM1_ESM.docx]

Sammendrag av studien om betydning av brukerutvalg tilknyttet rehabiliteringsinstitusjoner.

Bakgrunn

Brukermedvirkning fremheves som viktig for å få til best mulig behandling og rehabilitering for flest mulig. Brukermedvirkning på organisasjonsnivå er ofte organisert gjennom brukerutvalg med brukerrepresentanter som utvalgsmedlemmer, disse utvalgene er en integrert del av helseinstitusjonene i Norge.

Forskning viser at det er behov for mer kunnskap om hvordan brukerrepresentantene opplever sin medvirkning i forhold til organisering og innflytelse. Målet med denne studien var derfor å beskrive hvordan brukerrepresentanter i brukerutvalg opplever sine oppgaver, roller og påvirkning på beslutningsprosesser og utøvelse av rehabiliteringstjenester.

Metode

I denne studien benyttet vi den norske versjonen av et Kanadisk spørreskjema om brukermedvirkning, Evalueringsverktøy for Brukermedvirkning, EBNOR. Spørreskjemaet evaluerer praksis, organisering, innflytelse og påvirkning på brukermedvirkning i utvikling og utøvelse av helsetjenester. Totalt 47 brukerrepresentanter tilknyttet brukerutvalg ved rehabiliteringsparaplyorganisasjonene VIRKE og UNICARE i Norge svarte på 35 spørsmål om brukermedvirkning på organisasjonsnivå. Spørsmålene ble besvart ved å krysse av hvor enig eller uenig man var i de ulike utsagnene, fem av spørsmålene åpnet for fritekstbesvarelser.

Resultater

Resultatene viste at cirka 75 % var enige i at rehabiliteringsinstitusjonen samlet sett ble bedre som følge av brukermedvirkning. Hovedinntrykket er at de fleste brukerrepresentantene var fornøyde med hvordan rehabiliteringsinstitusjonene organiserte brukerutvalgene. Brukerrepresentanter i brukerutvalg opplevde likevel begrenset innflytelse og påvirkning.
