# Supplementary material for: Organisation, influence, and impact of patient advisory boards in rehabilitation institutions—an explorative cross-sectional study
Source: BMC Musculoskelet Disord. 2022 Aug 2;23:738. doi: 10.1186/s12891-022-05678-y (PMC9343240; doi:10.1186/s12891-022-05678-y)
Supplement: Supplementary file 2 — Additional file 2. Plain language summary in English. [file 12891_2022_5678_MOESM2_ESM.docx]

Summary related to the importance of patient advisory boards associated with rehabilitation institutions.

Background

Patient participation is emphasized as important in order to achieve the best possible treatment and rehabilitation for as many people as possible. Patient participation at the organisational level is often organized as patient advisory boards with patient representatives as board members, and represent an integral part of health institutions in Norway.

Research show that there is a need for more knowledge about how patient representatives experience their participation with regard to organisation and influence. The aim of this study was therefore to describe how patient representatives in patient advisory boards experience their tasks, roles, influence, and impact on decision-making processes and the delivery of rehabilitation services.

Method

In this study, we used the Norwegian version of the Canadian Public and Patient Engagement Evaluation Tool (PPEET), EBNOR in Norwegian. The questionnaire assesses practices, organization, influence, and impact of patient participation in the development, and delivery of health services. A total of 47 patient representatives associated with patient advisory boards registered by the rehabilitation umbrella organizations VIRKE and UNICARE in Norway answered 35 questions about patient participation at the organizational level. The questions were answered by choosing how much one agreed or disagreed with the various statements, five of the questions included free-text answers.

Results

The results showed that approximately 75% agreed that the rehabilitation institution as a whole improved as a result of patient participation. The findings further indicated that most patient representatives were satisfied with how the rehabilitation institutions organise the patient advisory boards. Nevertheless, patients who are members of patient advisory boards indicate limited influence and impact.
