# Supplementary material for: Organisation, influence, and impact of patient advisory boards in rehabilitation institutions—an explorative cross-sectional study
Source: BMC Musculoskelet Disord. 2022 Aug 2;23:738. doi: 10.1186/s12891-022-05678-y (PMC9343240; doi:10.1186/s12891-022-05678-y)
Supplement: Supplementary file 3 — Additional file 3. PPI using GRIPP2 – SF. [file 12891_2022_5678_MOESM3_ESM.docx]

**PPI in exploring the organisation, influence, and impact of patient advisory board representation in rehabilitation institutions using GRIPP 2-SFa.**

| Section and topic | Item |
| --- | --- |
| 1: Aim  Report the aim of the study | - Our research is conducted in close collaboration with “The Norwegian Federation of Organisations of Disabled People (FFO), a patient umbrella organization. The organisation consists of 82-member organizations (2017) of people with disabilities and chronic diseases, and a representative for patient advisory boards in rehabilitation. Together we have decided that our project is well suited to involve patient research partners throughout the project. |
|  |  |
| 2: Methods  Provide a clear description of the  methods used for PPI in the study | We aimed at following the European League Against Rheumatism (EULAR) recommendations for the inclusion of patient representatives in scientific projects (1).   - In our project, patient research partners were involved at different levels and stages, and had different roles (informant, adviser, collaborator and control). They are service users, related to health care services or work as controllers. The project is funded by DAM foundation, which consists of non–profit patient organizations. - Two actively engaged patient representatives have been part of the project group from the planning of research thesis. They have contributed to all parts of the development and implementation of the project. To ensure a degree of representativeness   the two actively engaged patient representatives were recruited from a patient umbrella organization (FFO) and from one of the included rehabilitation institutions.     - During the initial phase we clarified what kind of role the actively engaged patient research partners should have. In our project, they are seen as co-researchers and collaborators, but it is clarified from both parties we contribute the most where the competence is the best. They have participated in planning, discussion, interpretation, presentation, and publication of the results on an equal footing with the other members of the project group. They are seen as partners and co–authors. - All research partners will contribute in the dissemination process. The results will be presented in easily accessible lay journals, in peer-reviewed journals, through patient organizations, and in meetings with relevant stakeholders at local and national arenas, as well as communicated through mass media and social media. |
|  |  |
| 3: Results  Outcomes—Report the results of  PPI in the study, including both  positive and negative outcomes | PPI contributed to the study in several ways, including:   - Checked and commented on the survey itself, and on the invitation text to make ensure it was understandable. - Interpreted the results, especially results from free-text answers. - Contributed throughout the write-up phase for the results paper, including the final version, and they will contribute to the lay version. |
|  |  |
| 4: Discussion  Outcomes—Comment on the extent to  which PPI influenced the study overall.  Describe positive and negative effects | Patient and public involvement in this study worked well, and patient research partners had influence on important aspects of the study. However, some of the contributions were not implemented to keep the scope of the research.   - The patient partners were involved in determining the need for more knowledge on this specific topic. The two actively engaged research partners participated at several initial physical meeting, declaring the level of participation. Throughout the research process, the communication mostly worked through telephone and e-mails. However, ideally more physical meetings were on demand but were not feasible due to COVID – 19. The two research partners had different levels of research competence and therefore contributed differently. Nevertheless, we believe different competence and experience strengthen the representativeness of the contributions. |

References:

1. de Wit, M.P., et al., European League Against Rheumatism recommendations for the inclusion of patient representatives in scientific projects. Ann Rheum Dis, 2011. 70(5): p. 722-
